# Lower peripheral blood Toll-like receptor 3 expression is associated with an unfavorable outcome in severe COVID-19 patients

**DOI:** 10.1038/s41598-021-94624-4

**Published:** 2021-07-27

**Authors:** Maria Clara Saad Menezes, Alicia Dudy Müller Veiga, Thais Martins de Lima, Suely Kunimi Kubo Ariga, Hermes Vieira Barbeiro, Claudia de Lucena Moreira, Agnes Araujo Sardinha Pinto, Rodrigo Antonio Brandao, Julio Flavio Marchini, Julio Cesar Alencar, Lucas Oliveira Marino, Luz Marina Gomez, Niels Olsen Saraiva Camara, Heraldo P. Souza

**Affiliations:** 1 Emergency Medicine Department, Faculdade de Medicina da Universidade de São Paulo, Rua Dr. Arnaldo, 455, São Paulo, Brazil; 2 Department of Immunology, Institute of Biomedical Sciences, Universidade de São Paulo, São Paulo, Brazil

**Keywords:** Cytokines, Innate immunity, Biomarkers

## Abstract

The role of innate immunity in COVID-19 is not completely understood. Therefore, this study explored the impact of Severe Acute Respiratory Syndrome Coronavirus 2 (SARS-CoV-2) infection on the expression of Pattern Recognition Receptors (PRRs) in peripheral blood cells and their correlated cytokines. Seventy-nine patients with severe COVID-19 on admission, according to World Health Organization (WHO) classification, were divided into two groups: patients who needed mechanical ventilation and/or deceased (SEVERE, n = 50) and patients who used supplementary oxygen but not mechanical ventilation and survived (MILD, n = 29); a control group (CONTROL, n = 17) was also enrolled. In the peripheral blood, gene expression (mRNA) of Toll-like receptors (TLRs) 3, 4, 7, 8, and 9, retinoic-acid inducible gene I (RIGI), NOD-like receptor family pyrin domain containing 3 (NLRP3), interferon alpha (IFN-α), interferon beta (IFN-β), interferon gamma (IFN-γ), interferon lambda (IFN-λ), pro-interleukin(IL)-1β (pro-IL-1β), and IL-18 was determined on admission, between 5–9 days, and between 10–15 days. Circulating cytokines in plasma were also measured. When compared to the COVID-19 MILD group, the COVID-19 SEVERE group had lower expression of TLR3 and overexpression of TLR4.

Since the beginning of the current pandemic, progress has been made in our understanding of COVID-19. The disease's clinical and epidemiological aspects^1,2^, and methods to identify patients at higher risk of respiratory failure have been developed^3–5^. On the other hand, our knowledge about the immune response to SARS-CoV-2 is incomplete^6^, although important findings were reported, related to interferon secretion^7^ and the role of distinct immune cell types in the disease pathophysiology^8^.

Like other respiratory coronaviruses, SARS-CoV-2 is transmitted primarily via respiratory droplets^9^. Once in the lower respiratory tract, it utilizes the angiotensin-converting enzyme II (ACEII) to enter the intracellular environment^10^. This process is followed by the production of cytokines, infiltration of inflammatory cells in the alveolar space^11^, leading to respiratory failure and a systemic inflammatory response^12^. Increased circulating inflammatory markers are found in patients at the most severe spectrum of the disease, including C-Reactive Protein, procalcitonin, and ferritin^13^. Moreover, elevated IL-6 and tumor necrosis factor-alpha (TNF-α) serum levels at presentation are strong independent predictors of disease severity and survival^11^.

The triggering mechanisms for this enhanced inflammatory reaction are not yet clear; this response is probably initiated by the activation of PRRs, which are responsible for the detection of pathogen-associated and danger-associated molecular patterns^14^. Virus-responsive PRRs comprise a series of receptors, like the TLRs^15^, NLRs^16^, and others. It is known that endosomal TLRs 3 and 7^17^ and RIG-I^18^ are involved in recognizing RNA viruses, and they were reported to be activated in COVID-19^19,20^. Other TLRs (4, 8, and 9) also collaborate in the antiviral response or in assembling an inflammatory response to eliminate the aggressor^21^.

Therefore, modulating PRRs expression may be a manner of controlling the innate immune response, as observed in patients with bacterial sepsis^22^. This study explored the impact that SARS-CoV-2 infection could have in the peripheral blood expression of PRRs and, consequently, in cytokines with antiviral activity. Here, we demonstrate that lower expression of TLR-3 and an enhanced expression of TLR-4 are associated with an unfavorable outcome in COVID-19 patients.

## Methods

### Study design and ethics statement

This is a prospective cohort study, with patients enrolled at the Emergency Department of the Hospital das Clinicas da Faculdade de Medicina da Universidade de São Paulo, São Paulo, Brazil—a tertiary hospital designated to assist only patients with severe COVID-19 during the pandemics.

The protocol was approved by the institutional Ethics Committee, under number CAAE: 30417520.0.0000.0068. All patients, or their legal representatives, signed the informed consent. All experiments were performed following relevant guidelines and regulations.

### Enrollment criteria

Seventy-nine patients admitted to the Emergency Department with severe COVID-19 on admission, according to WHO guidelines (Supplementary Table 1)^23^, were enrolled. COVID-19 was diagnosed by a positive SARS-CoV-2 RT-PCR test in nasopharyngeal and throat swabs and by typical chest CT-scan findings. All enrolled patients had hypoxemia (as defined by peripheral oxygen saturation less than 92%) and were using supplemental oxygen. Patients under 18 years old, pregnant women, and patients in end-of-life protocols were not included.

### Initial assessment

All patients were evaluated and treated according to the Institution's protocol. They were submitted to CT, routine laboratory tests, and an additional 20 ml blood sample was collected in a tube with trisodium citrate that was immediately sent to the laboratory. The whole blood was separated by centrifugation, and small aliquots of leukocytes and plasma were stored at  − 80 °C until the measurements.

### Clinical outcomes and study groups

After emergency department assessment, patients were transferred to hospital wards or intensive care units. Clinical, radiologic, and routine laboratory tests were obtained from the electronic medical records.

Enrolled COVID-19 patients (n = 79), had severe COVID-19 on admission, according to WHO guidelines^21^ and were further classified into two groups: patients who needed mechanical ventilation and/or deceased (SEVERE, n = 50) and patients who used supplementary oxygen but not mechanical ventilation and survived (MILD, n = 29). Demographic and clinical data from these groups are shown in Table 1. A control group (CONTROL, n = 17) composed of healthy patients with no history of COVID-19 or active respiratory disease and similar demographic characteristics was also enrolled.

**Table 1:** Demographic and clinical characteristics of the patients enrolled in the study.

	MILD		SEVERE		
	n	%	n	%	*p*
Number	29		50		
Male sex	17	58.6	35	70.0	0.694*
Mortality	**0**	**0.0**	**41**	**82.0**	0.000*
Hypertension	17	58.6	30	60.0	1.000*
Diabetes	11	37.9	25	50.0	0.782*
Obesity	3	10.3	12	24.0	0.527*
Chronic kidney disease	3	10.3	4	8.0	0.989*
Chronic heart disease	6	20.7	10	20.0	1.000*
Chronic pulmonary disease	2	6.9	7	14.0	0.821*
Immunosuppressive drugs	12	41.4	33	66.0	0.209*

	Median	IQR	Median	IQR	*p*
Age	61	(51–73)	63.5	(55.2–69)	0.8786**
Days of symptoms	7	(4–10)	8	(5–11)	0.269**
Leukocytes	**7.17**	**(5.77–9.57)**	**10.6**	**(7.52–12.8)**	0.014**
Neutrophils	**6.11**	**(4.3–8.14)**	**9.15**	**(6.4–11.4)**	0.004**
Lymphocytes	0.99	(0.68–1.59)	0.915	(0.57–1.26)	0.261**
CRP	**102**	**(47.6–161)**	**156**	**(85.9–224)**	0.0197**

Variables are expressed as number (%) or median (interquartile range). Bolded values indicated variables with statistically significant associations (significance level of 0.05).

*P values were calculated using the chi-square test in R software (version 4.0.3 for macOS).

**P values were calculated using the nonparametric Mann–Whitney test in R software (version 4.0.3 for macOS), that tests for trend across ordered groups.

To determine the distribution of TLR3 in different types of blood leukocytes, another set of 20 patients were included in the study, ten with our criteria for SEVERE disease and ten with MILD disease. From these additional patients, blood cells were separated by the Ficoll method in mononuclear and polymorphonuclear cells. RNA was further obtained from these cells for analyses, as described below.

### RNA isolation and RT-PCR

Samples with whole white blood cells were thawed and diluted in a solution with Trizol (Thermo Fisher Scientific) and DNAse, according to the supplier's instructions. Extracted RNA was quantified by a photometer (Eppendorf) at 260 nm wavelength. The quantitative real-time polymerase chain reaction was carried out with SuperScript III Platinum One-Step qRT-PCR Kit with ROX (Thermo Fisher Scientific - #11745100) in thermocycler Step One Real-Time PCR System (Applied Biosystems) for TLR 3, 4, 7, 8 and 9, RIGI, NLRP3, IL18, IFN-α, IFN-β, IFN-γ, IFN-λ and pro-IL-1β. B2M was used as a housekeeping gene. Primer sequences (Supplementary Table 2) were obtained from the PrimerBank (https://pga.mgh.harvard.edu/primerbank/). Gene expression was quantified by the 2-ΔΔCT method.

### Plasma cytokine measurement

Plasma samples were thawed, and cytokines (IFN-γ, IL-6, IL-10, IL-1β, TNF-α and monocyte chemoattractant protein-1 [MCP-1]) were measured by Human Cytokine/Chemokine Magnetic Bead Panel protocol from the Milliplex®, according to the manufacturer’s instructions.

### Statistics

Continuous variables were submitted to the Shapiro-Wilkins test for normality. When distribution was normal, Student's t-test was used to compare the medians, and results were presented as mean ± standard error of the mean (SEM). Kruskal Wallis test with Dunn's correction and Mann–Whitney test were used when data were not normally distributed, and data are presented as median and interquartile range. For categorical variables, the chi-square test was performed. Pearson correlation test was used to measure the degree of association between two variables. The significance level was set at 0.05. All statistical analyses were performed with R software (version 4.0.3 for macOS).

## Results

### Patients demographic and clinical characteristics

There was no difference between MILD and SEVERE groups regarding demographic and clinical features, and routine laboratory tests, except circulating C-reactive protein, leukocytes and neutrophils, which were higher in the SEVERE group (Table 1).

### Innate immunity receptors and cytokines at enrollment

**Figure 1:**
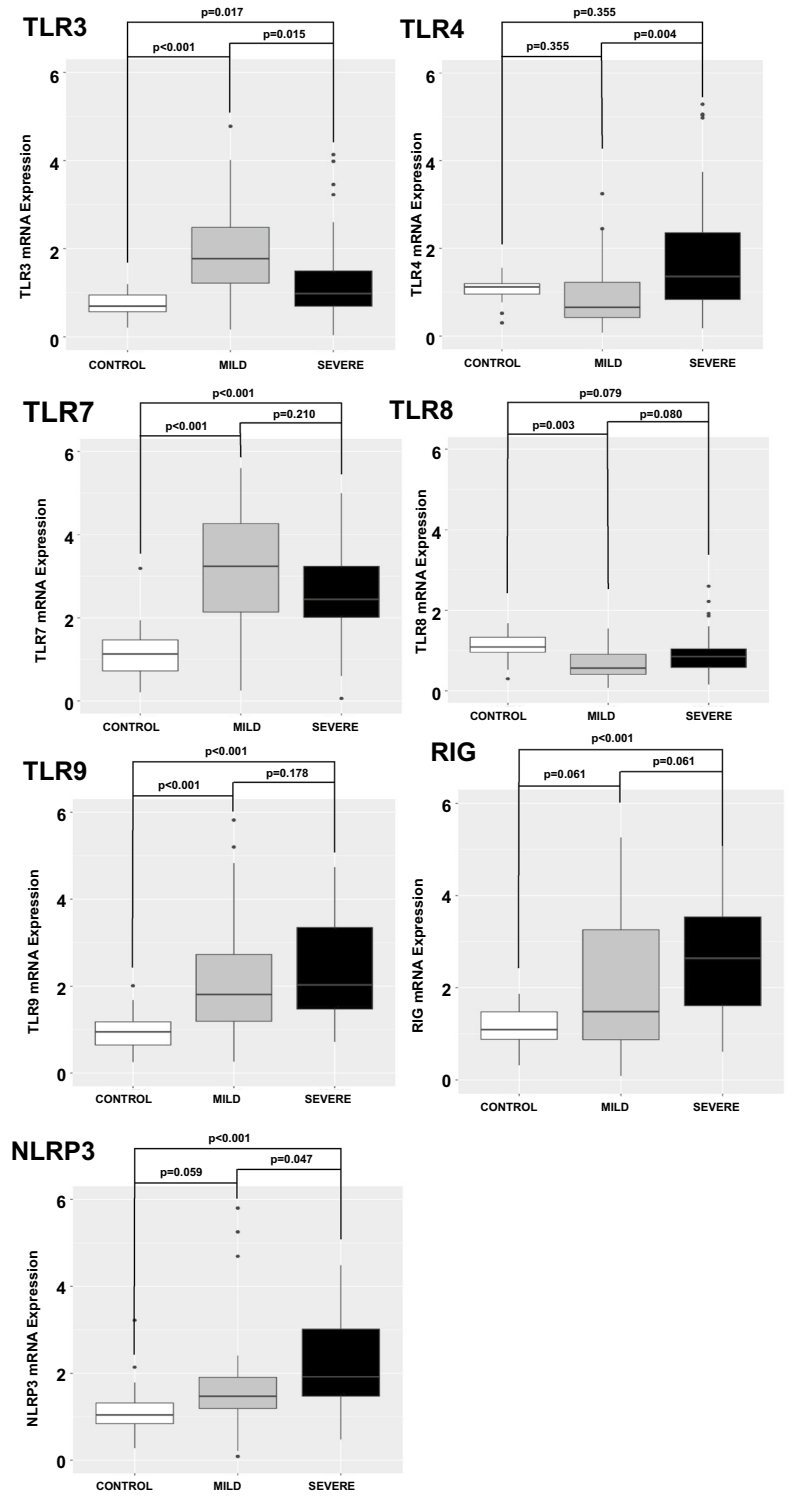
Quantitative mRNA of Pattern Recognition Receptors (PRRs) in circulating leukocytes of patients with COVID-19 on admission day. Expression of PRRs (mRNA) was measured in circulating leukocytes. Compared to the CONTROL group (N = 17 patients), increased mRNA transcription was observed for TLRs 3, 7, 9, RIGI, and NLRP3 in COVID-19 patients, while TLR8 was decreased. No significant difference in mRNA transcription for TLRs 7, 8 and 9, RIG, or NLRP3 was observed between COVID-19 MILD (N = 29 patients) and COVID-19 SEVERE (N = 50 patients) groups. On the other hand, TLR3 mRNA was decreased, while TLR4 mRNA was increased in COVID-19 SEVERE group when compared to COVID-19 MILD group. P values were calculated using the nonparametric Kruskal–Wallis test in R software (version 4.0.3 for macOS).

Compared to the CONTROL group, increased mRNA transcription was observed for TLRs 3, 7, 9, RIGI, and NLRP3 in the entire COVID-19 group, while TLR8 was decreased. No significant difference in mRNA transcription for TLRs 7, 8 and 9, RIGI, or NLRP3 was observed between the COVID-19 MILD and the COVID-19 SEVERE groups. On the other hand, TLR3 mRNA was decreased, while TLR4 mRNA was increased in the COVID-19 SEVERE group when compared to the COVID-19 MILD group (Fig. 1).

In a separate set of patients (COVID-19 MILD N = 10; COVID-19 SEVERE N = 10), differences in TLR3 expression between distinct cell types were investigated. TLR3 mRNA mean expression in polymorphonuclear and mononuclear cells in the COVID-19 MILD group was 2.06 and 1.94, respectively (*p* 0.945; Supplementary Table 3). TLR3 mRNA mean expression in polymorphonuclear and mononuclear cells in the COVID-19 SEVERE group was 1.82 and 1.77 respectively (*p* 0.931; Supplementary Table 3).TLR3 mRNA mean expression in polymorphonuclear was 2.06 in the MILD group and 1.82 in the SEVERE group however not statistically significant (p 0.821; Supplementary Table 3). TLR3 mRNA mean expression in mononuclear cells was 1.94 in the MILD group and 1.77 in the SEVERE group (*p* 0.866; Supplementary Table 3).

**Figure 2:**
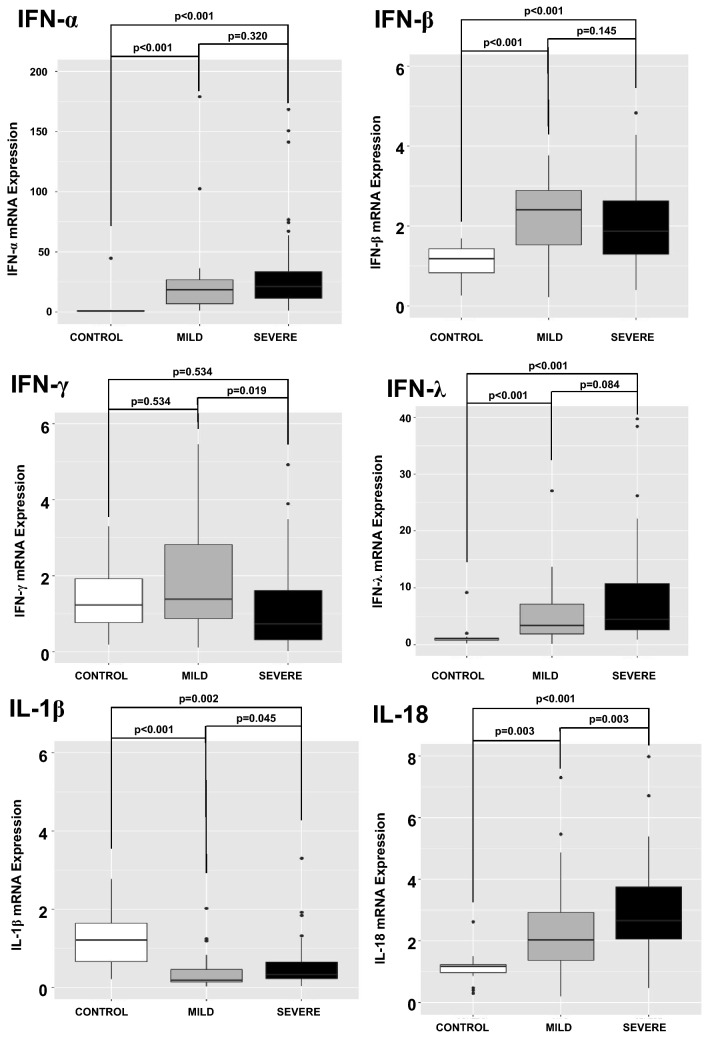
Quantitative mRNA of cytokines in circulating leukocytes of patients with COVID-19 on admission day. Expression of mRNAs of cytokines was measured in circulating leukocytes. IFN-α, IFN-β, IFN-λ, and IL-18 were increased in COVID-19 patients (N = 79 patients; 29 MILD and 50 SEVERE) when compared to CONTROL subjects (N = 17 patients). Pro-IL-1β mRNA was decreased in COVID-19 patients when compared to CONTROL subjects. No difference in IFN-γ expression was observed between CONTROL and COVID-19 patients. P values were calculated using the nonparametric Kruskal–Wallis test in R software (version 4.0.3 for macOS).

An analysis of cytokines mRNA transcription was also performed. IFN-α, IFN-β, IFN-λ, and IL-18 were increased in COVID-19 patients when compared to CONTROL subjects. Pro-IL-1β mRNA was decreased in COVID-19 patients when compared to CONTROL subjects. No difference in IFN-γ mRNA was observed between CONTROL and COVID-19 patients (Fig. 2).

IFN-γ mRNA expression was lower in the COVID-19 SEVERE when compared to COVID-19 MILD. IL-18 transcription was higher in the COVID-19 SEVERE when compared to COVID-19 MILD (Fig. 2).

**Figure 3:**
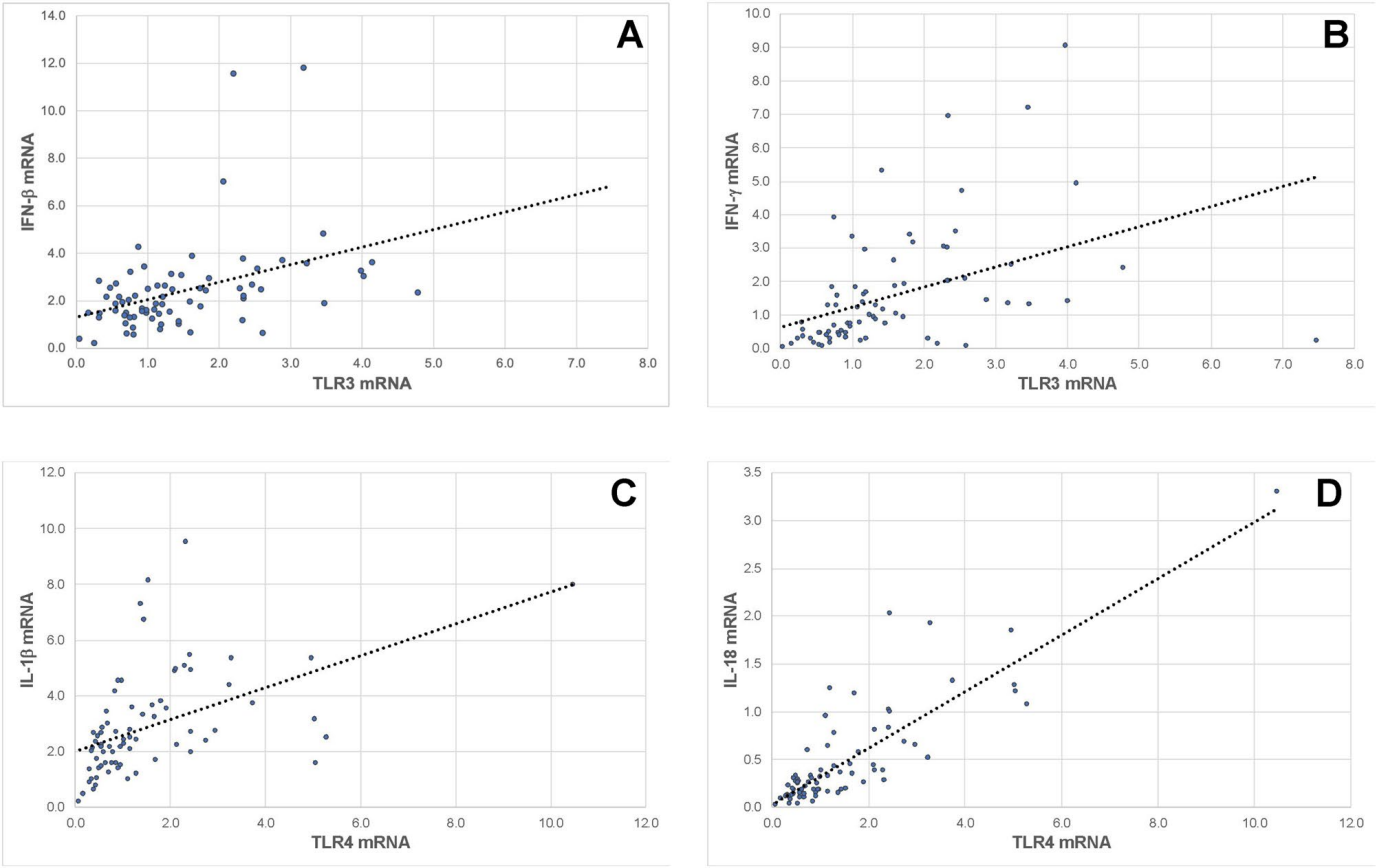
Association between Toll-like Receptors (TLRs) 3 and 4 and cytokine expression. The expression of IFN-γ and IFN-β was positively associated with TLR3 expression (Panels **A** and **B**) with R^2^ = 0.263 and 0.282, respectively. Expression of pro-IL-1β and IL-18 was positively associated with the expression of TLR4 (Panels C and D), with R^2^ = 0.239 and 0.713, respectively. Pearson correlation coefficient was used in R software (version 4.0.3 for macOS).

The association between the TLR3 and TLR4 mRNA expression and the antiviral cytokines was investigated. There was a positive correlation between IFN-β and IFN-γ mRNA expression and TLR3 mRNA expression (Fig. 3, Panels A and B), and a positive correlation between pro-IL-1β and IL-18 and TLR4 expression (Fig. 3, Panels C and D).

### Innate immunity receptors during the hospital stay

**Figure 4:**
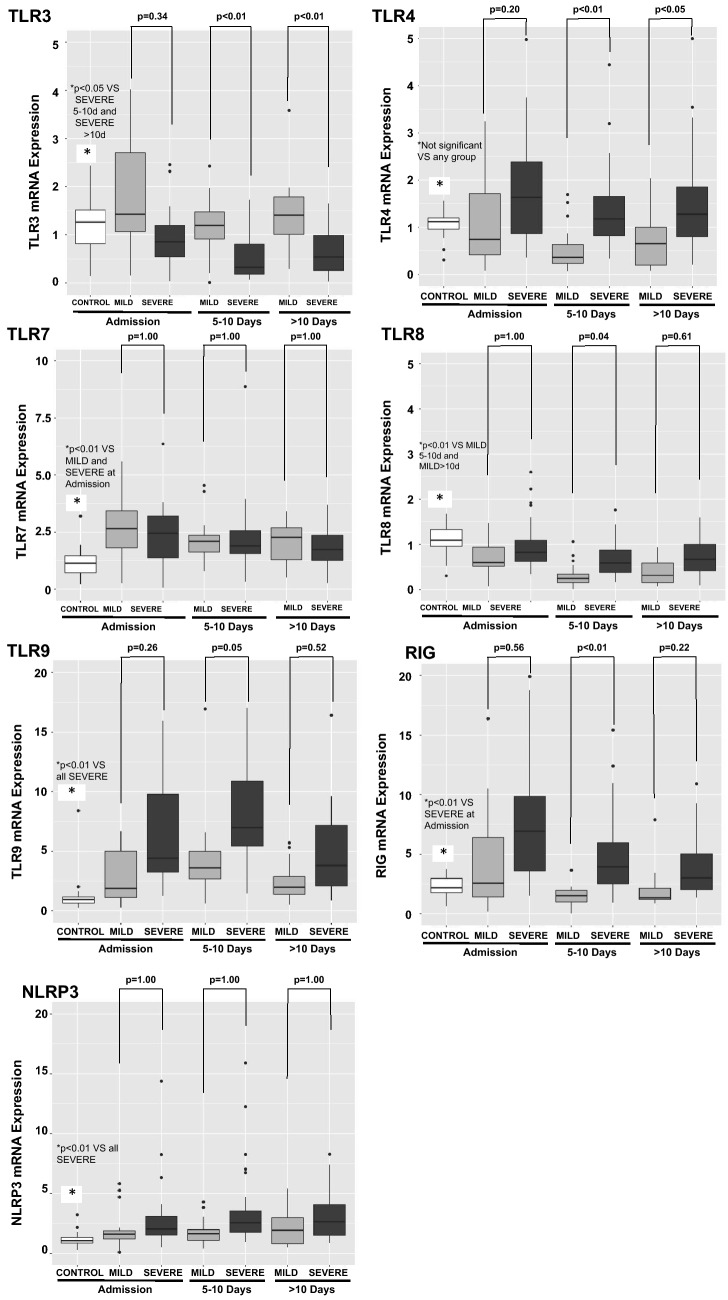
Quantitative mRNA of Pattern Recognition Receptors (PRRs) in circulating leukocytes of patients with COVID-19 during hospital stay. Expression of PRRs (mRNA) was measured in circulating leukocytes. Blood samples were collected every five days as feasible during hospitalization. The difference between COVID-19 MILD (N = 17 patients) and COVID-19 SEVERE (N = 24 patients) TLR3 and TLR4 mRNA expression at admission widened during follow up. Controls are also shown (17 patients). *P* values were calculated using the nonparametric Kruskal–Wallis test in R software (version 4.0.3 for macOS).

**Figure 5:**
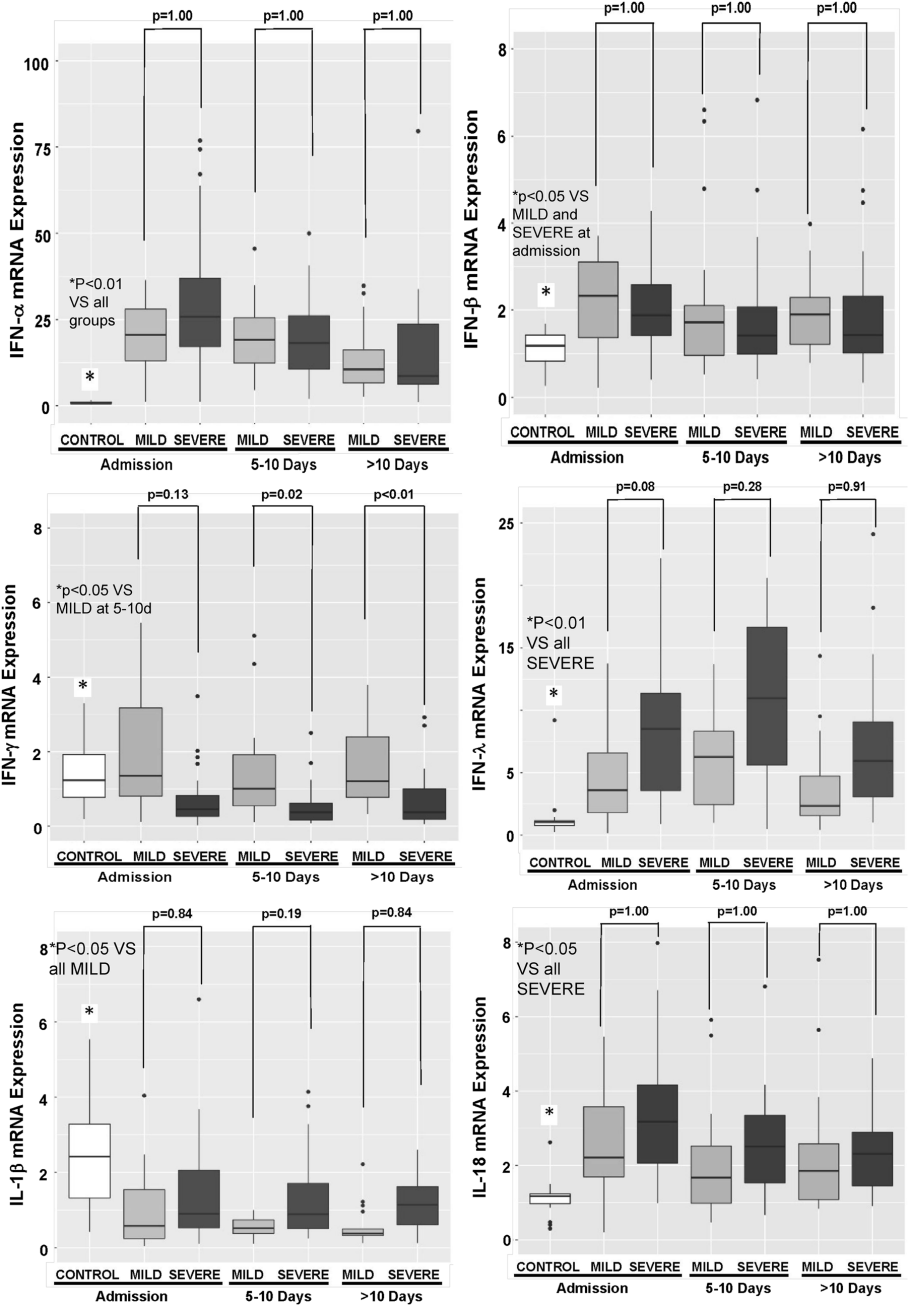
Quantitative mRNA of cytokines in circulating leukocytes of patients with COVID-19 during hospital stay. Expression of cytokines (mRNA) was measured in circulating leukocytes. Blood samples were collected every five days as feasible during hospitalization. IFN-γ mRNA expression was lower in COVID-19 SEVERE (N = 24 patients) in comparison to COVID-19 MILD (N = 17 patients), and widened during the hospital internment. Controls are also shown (17 patients). *P* values were calculated using the nonparametric Kruskal–Wallis test in R software (version 4.0.3 for macOS).

During hospitalization, blood samples were collected every five days as feasible. The difference between COVID-19 MILD and COVID-19 SEVERE TLR3 and TLR4 mRNA expression at admission widened during follow-up. The differential expression of IFN-γ between COVID-19 MILD and COVID-19 SEVERE also widened (Figs. 4 and 5).

**Figure 6:**
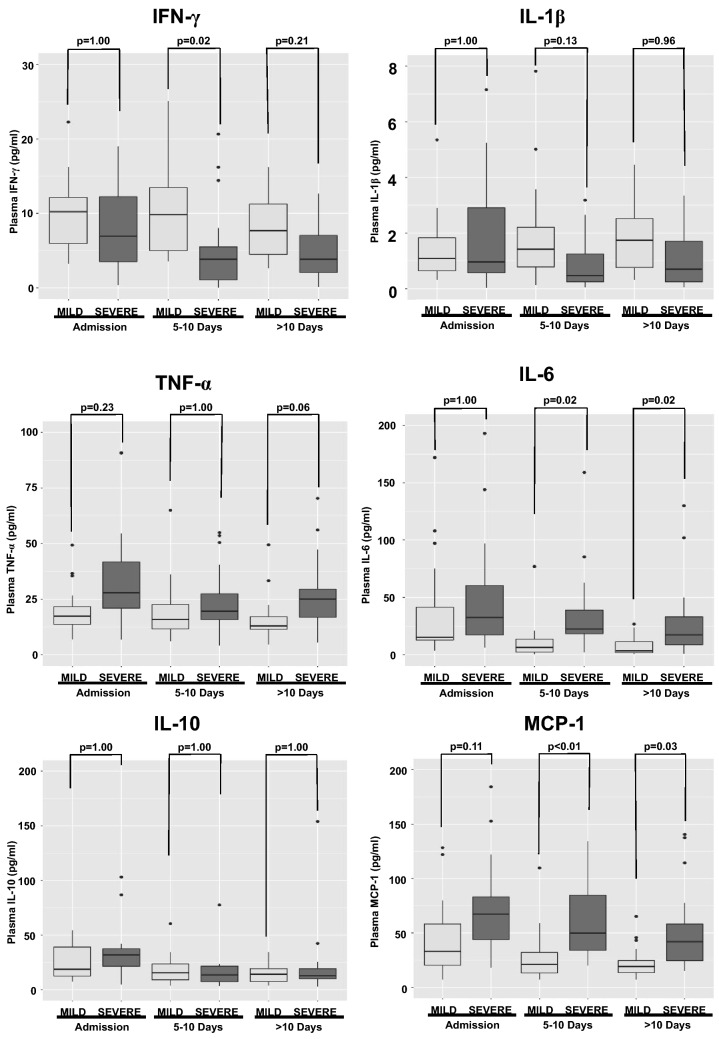
Serum cytokine levels during hospitalization. Circulating cytokines in plasma from the COVID-19 patients was also measured, and the profile was similar to that observed for mRNA. IFN-γ was lower in the SEVERE COVID-19 group (N = 50 patients) when compared to COVID-19 MILD (N = 29 patients), while pro-inflammatory cytokine levels (IL-6, TNF-α, and MCP-1) were higher. *P* values were calculated using the nonparametric Kruskal–Wallis test in R software (version 4.0.3 for macOS).

Circulating cytokines in plasma from the COVID-19 patients were also measured, and their profile was similar to that observed for mRNA. IFN-γ was lower in the SEVERE COVID-19 group when compared to COVID-19 MILD, while pro-inflammatory cytokine levels (IL-6, TNF-α, and MCP-1) were higher (Fig. 6).

## Discussion

Sensing the invading pathogens by PRRs is the beginning of the antiviral defense line^24^. In this study, we evaluated the gene expression of PRRs engaged in antiviral response (TLRs 3, 7, 8, 9, RIGI and NLRP3).

Most PRRs were upregulated in COVID-19 patients compared to CONTROL. The only exception was TLR8. It is known that 1,25-dihydroxyvitamin D3 may impair TLR8 expression^25^, and vitamin D deficiency was associated with susceptibility to COVID-19^26^. However, we did not pursue this line of reasoning in this study since our primary interest was to identify the differential expression of PRRs when comparing COVID-19 MILD and COVID-19 SEVERE (TLR8 expression did not differ between those groups). When comparing COVID-19 MILD and COVID-19 SEVERE groups, a decreased expression of TLR3, accompanied by a deficient IFN-γ and an enhanced TLR4 expression were associated with an unfavorable outcome.

TLR3 has been described as an integral part of the immune response to several double-stranded RNA viruses^27^; however, it was demonstrated that single-stranded RNA segments harboring stem structures with bulge/internal loops are also potent TLR3 agonists^28^. Moreover, TLR3-/-mice are more susceptible to SARS-CoV infection than wild-type animals^17^, and a porcine coronavirus was able to activate this receptor^29^.

In humans, Zhang et al. found autosomal-dominant deficiencies in patients with life-threatening COVID-19 that included mutations in the TLR3 gene^30^. In the same study, the authors showed that TLR3-/-fibroblasts were more susceptible to SARS-CoV-2 infection in vitro. These data point out a prominent role for TLR3 in the immune response to coronavirus also in humans.

The distinct TLR3 expression between MILD and SEVERE groups could be secondary to increased neutrophils in the SEVERE group (9.15 VS 6.11, *p* 0.004, Supplementary Table 3) as in peripheral blood there is evidence suggesting that TLR3 expression is enhanced in T-cells^31^. Consequently, the lower TLR3 expression in the COVID-19 SEVERE group could reflect a diminished fraction of lymphocytes in their peripheral blood samples. However, the evidence mentioned above^31^ was obtained from naïve cells. As observed in Fig. 1, peripheral blood cells from COVID-19 patients present a distinct mRNA profile, with increased expression of all PRRs compared to cells from healthy subjects. Hence, an investigation of TLR3 expression among mononuclear and polymorphonuclear cells in the peripheral blood of COVID-19 patients was performed (Supplementary Table 3). The results demonstrated that TLR3 expression is similar in mononuclear cells and polymorphonuclear cells. TLR3 expression was slightly higher in the MILD group than in the SEVERE group but not statistically significant which may be related to the small number of samples evaluated in the experiment. Altogether, these data suggest that lower TLR3 expression in SEVERE patients was not related only to the increased number of neutrophils in these individuals. However, further studies are needed to find the source of the difference in TLR3 expression if it is due to an overall decrease in TLR3 expression in all cell subsets or a specific cell subset.

Also, there was a statistically significant, positive correlation between TLR3 and IFN-γ expression. Engaging TLR3 by double-stranded RNA viruses has been shown to induce a robust cytokine response, including IFN-γ^32^. However, this relationship is complex since IFN-γ can also increase TLR3 expression via signal transducer and activator of transcription 1 (STAT1) in cultured normal human epidermal keratinocytes^33^. Proving any causal relationship between TLR3 and IFN-γ expression requires a different experimental model.

An enhanced TLR4 expression was associated with the COVID-19 SEVERE group when compared to COVID-19 MILD. TLR4 recognizes lipopolysaccharide from gram-negative bacteria^34^ but in silico studies suggested that it can also contribute to the response to the SARS-CoV-2 infection^35^.

Contrary to TLR3, the mRNA transcription for TLR4 was higher in the COVID-19 SEVERE group. This finding may represent a compensatory response by leukocytes when TLR3 expression is deficient. TLR3 and TLR4 often work synergistically against a viral challenge, utilizing the same network of intracellular signaling pathways^36^. Therefore, it seems reasonable to hypothesize that an impaired TLR3 would lead to a compensatory enhancement in TLR4 mRNA translation. The increased TLR4 mRNA expression may lead to a prominent inflammatory response, characteristic of the life-threatening COVID-19 disease.

Some limitations must be considered. Gene transcription was measured, but not protein expression. Also, although some correlations between PRRs and cytokines were described, as this is an observational study it is not an appropriate method to uncover causal relationships. On the other hand, the data was obtained from real patients and allowed in vivo measurements. Moreover, differences in peripheral blood cell type subsets between groups can interfere in the differences of PRRs mRNA expression measured. Finally, only the cells in the peripheral blood were analyzed. At baseline, the TLR expression profile among blood and lung resident cells is not identical^31^. In the present study, the inflammatory cells that migrated to the alveoli during COVID-19 induced ARDS were not evaluated. Conclusions about the expression of PRRs inside the lungs during COVID-19 cannot be inferred.

In summary, our research indicated that patients with an unfavorable outcome presented lower TLR3 expression and enhanced expression of TLR4 which may be a compensatory mechanism that leads to a prominent inflammatory response that is characteristic of severely ill COVID-19 patients. Our data support the possibility of using TLR3 expression to identify patients at risk of life-threatening COVID-19. Further research is necessary to understand the potential of modulating TLR3 expression as a method to prevent or treat COVID-19.

## Supplementary Information

Supplementary Information.

## Data Availability

The datasets generated and/or analyzed during the current study are available from the corresponding author on reasonable request.

## Abbreviations

ACEII: Angiotensin-converting enzyme II
IFN-α: Interferon alpha
IFN-β: Interferon beta
IFN-γ: Interferon gamma
IFN-λ: Interferon lambda
IL: Interleukin
MCP-1: Monocyte chemoattractant protein-1
NLRP3: NOD-like receptor family pyrin domain containing 3
pro-IL-1β: Pro-interleukin-1β
PRRs: Pattern recognition receptors
RIGI: Retinoic-acid inducible gene I
SARS-CoV-2: Severe acute respiratory syndrome coronavirus 2
STAT1: Signal transducer and activator of transcription 1
TLRs: Toll-like receptors
TNF-α: Tumor necrosis factor alpha
WHO: World Health Organization

## References

[1] Docherty AB (2020). Features of 20 133 UK patients in hospital with covid-19 using the ISARIC WHO clinical characterisation protocol: Prospective observational cohort study. BMJ.

[2] Ranzani OT (2021). Characterisation of the first 250,000 hospital admissions for COVID-19 in Brazil: A retrospective analysis of nationwide data. Lancet Respir. Med..

[3] de Alencar JCG (2021). Lung ultrasound score predicts outcomes in COVID-19 patients admitted to the emergency department. Ann. Intensive Care.

[4] Antunez Muiños PJ (2021). The COVID-19 lab score: an accurate dynamic tool to predict in-hospital outcomes in COVID-19 patients. Sci. Rep..

[5] Wynants L (2020). Prediction models for diagnosis and prognosis of covid-19 infection: Systematic review and critical appraisal. BMJ.

[6] Schultze JL, Aschenbrenner AC (2021). COVID-19 and the human innate immune system. Cell.

[7] Galani I-E (2021). Untuned antiviral immunity in COVID-19 revealed by temporal type I/III interferon patterns and flu comparison. Nat. Immunol..

[8] Daamen AR (2021). Comprehensive transcriptomic analysis of COVID-19 blood, lung, and airway. Sci. Rep..

[9] Anfinrud P, Stadnytskyi V, Bax CE, Bax A (2020). Visualizing speech-generated oral fluid droplets with laser light scattering. N. Engl. J. Med..

[10] Letko M, Marzi A, Munster V (2020). Functional assessment of cell entry and receptor usage for SARS-CoV-2 and other lineage B betacoronaviruses. Nat. Microbiol..

[11] Del Valle DM (2020). An inflammatory cytokine signature predicts COVID-19 severity and survival. Nat. Med..

[12] Perier F (2020). Effect of PEEP and proning on ventilation and perfusion in COVID-19 ARDS. Am. J. Respir. Crit. Care Med..

[13] Brandão Neto RA (2021). Mortality and other outcomes of patients with coronavirus disease pneumonia admitted to the emergency department: A prospective observational Brazilian study. PLoS ONE.

[14] Mogensen TH (2009). Pathogen recognition and inflammatory signaling in innate immune defenses. Clin. Microbiol. Rev..

[15] Kawai T, Akira S (2010). The role of pattern-recognition receptors in innate immunity: update on toll-like receptors. Nat. Immunol..

[16] Velloso FJ, Trombetta-Lima M, Anschau V, Sogayar MC, Correa RG (2019). NOD-like receptors: Major players (and targets) in the interface between innate immunity and cancer. Biosci. Rep..

[17] Totura AL (2015). Toll-like receptor 3 signaling via TRIF contributes to a protective innate immune response to severe acute respiratory syndrome coronavirus infection. MBio.

[18] Yoneyama M (2004). The RNA helicase RIG-I has an essential function in double-stranded RNA-induced innate antiviral responses. Nat. Immunol..

[19] Lévy R (2021). Correction to: IFN-α2a therapy in two patients with inborn errors of TLR3 and IRF3 infected with SARS-CoV-2. J. Clin. Immunol..

[20] Chen K (2020). SARS-CoV-2 nucleocapsid protein interacts with RIG-I and represses RIG-mediated IFN-β production. Viruses.

[21] Miyake K (2018). Mechanisms controlling nucleic acid-sensing Toll-like receptors. Int. Immunol..

[22] Salomao R (2012). Bacterial sensing, cell signaling, and modulation of the immune response during sepsis. Shock.

[23] WHO. Clinical Management of Covid-19—Interim Guidance. https://www.who.int/publications/i/item/clinical-management-of-covid-19. Accessed 7 May 2021.

[24] Thompson J, Iwasaki A (2008). Toll-like receptors regulation of viral infection and disease. Adv. Drug Deliv. Rev..

[25] Li B (2013). 1,25-dihydroxyvitamin D3 suppresses TLR8 expression and TLR8-mediated inflammatory responses in monocytes in vitro and experimental autoimmune encephalomyelitis in vivo. PLoS ONE.

[26] Meltzer DO (2020). Association of vitamin D status and other clinical characteristics with COVID-19 test results. JAMA Netw. Open.

[27] Fitzgerald KA, Kagan JC (2020). Toll-like receptors and the control of immunity. Cell.

[28] Tatematsu M, Nishikawa F, Seya T, Matsumoto M (2013). Toll-like receptor 3 recognizes incomplete stem structures in single-stranded viral RNA. Nat. Commun..

[29] Xu Z (2019). Porcine deltacoronavirus induces TLR3, IL-12, IFN-α, IFN-β and PKR mRNA expression in infected Peyer’s patches in vivo. Vet. Microbiol..

[30] Zhang, Q. *et al.* Inborn errors of type I IFN immunity in patients with life-threatening COVID-19. *Science* **370**, (2020). 10.1126/science.abd4570 PMC7857407 32972995

[31] Uhlen M, Karlsson MJ, Zhong W, Tebani A, Pou C (2019). A genome-wide transcriptomic analysis of protein-coding genes in human blood cells. Science.

[32] Alexopoulou L, Holt AC, Medzhitov R, Flavell RA (2001). Recognition of double-stranded RNA and activation of NF-kappaB by Toll-like receptor 3. Nature.

[33] Kajita AI (2015). Interferon-gamma enhances TLR3 expression and anti-viral activity in keratinocytes. J. Invest. Dermatol..

[34] Takeuchi O (1999). Differential roles of TLR2 and TLR4 in recognition of gram-negative and gram-positive bacterial cell wall components. Immunity.

[35] Choudhury A, Mukherjee S (2020). In silico studies on the comparative characterization of the interactions of SARS-CoV-2 spike glycoprotein with ACE-2 receptor homologs and human TLRs. J. Med. Virol..

[36] Doyle S (2002). IRF3 mediates a TLR3/TLR4-specific antiviral gene program. Immunity.

