# Supplementary material for: Lower peripheral blood Toll-like receptor 3 expression is associated with an unfavorable outcome in severe COVID-19 patients
Source: Sci Rep. 2021 Jul 27;11:15223. doi: 10.1038/s41598-021-94624-4 (PMC8316546; doi:10.1038/s41598-021-94624-4)
Supplement: Supplementary file 1 — Supplementary Information. [file 41598_2021_94624_MOESM1_ESM.docx]

Supplementary table 1: Severe COVID-19 disease according to World Health Organization guidelines^21^

| COVID-19 disease severity - World Health Organization | |
| --- | --- |
| Mild disease | Symptomatic patients meeting the case definition for COVID-19 without evidence of viral pneumonia or hypoxia. |
| Moderate disease | Adolescent or adult with clinical signs of pneumonia (fever, cough, dyspnoea, fast breathing) but no signs of severe pneumonia, including SpO2 ≥ 90% on room air. |
| Severe disease | Adolescent or adult with clinical signs of pneumonia (fever, cough,  dyspnoea, fast breathing) plus one of the following: respiratory rate > 30  breaths/min; severe respiratory distress; or SpO2 < 90% on room air |

Supplementary table 2: Primers sequences and annealing temperature

| Gene | Primer | Size  (bp) | Temp. |
| --- | --- | --- | --- |
| B2M | F: 5’- GATGAGTATGCCTGCCGTGTG -3’  R: 5’- CAATCCAAATGCGGCATCT -3’ | 61 | 60 ºC |
| IFN-α | F: 5’- TGATCTCCCTGAGACCCACAGC -3’  R: 5’- CTGCTGGATCAGCTCATGGAGG -3’ | 187 | 57 ºC |
| IFN-β | F: 5’- GCTTGGATTCCTACAAAGAAGCA -3’  R: 5’- ATAGATGGTCAATGCGGCGTC -3’ | 88 | 60 ºC |
| IFN-γ | F: 5’- TTCAGCTCTGCATCGTTTTG -3’  R: 5’- TCTTTTGGATGCTCTGGTCA -3’ | 198 | 55 ºC |
| IFN-lambda1 | F:5’- GCCTCCTCACGCGAGACCTC-3’  R:5’- GGAGTAGGGCTCAGCGCATA -3’ | 157 | 60 ºC |
| IL-1β | F:5’- GGGCCTCAAGGAAAAGAATC -3’  R:5’- TTCTGCTTGAGAGGTGCTTGA-3’ | 205 | 55 ºC |
| IL-18 | F:5’ – TCTTCATTGACCAAGGAAATCGG -3’  R:5’ – TCCGGGGTGCATTATCTCTAC -3’ | 75 | 60 ºC |
| NLRP3 | F: 5’- CGTGAGTCCCATTAAGATGGAGT -3’  R: 5’- CCCGACAGTGGATATAGAACAGA -3’ | 191 | 60 ºC |
| RIG | F:5’- TGCGAATCAGATCCCAGTGTA -3’  R:5’- TGCCTGTAACTCTATACCCATGT -3’ | 83 | 60 ºC |
| TLR3 | F:5’ – CAAACACAAGCATTCGGAATCTG -3’  R:5’- AAGGAATCGTTACCAACCACATT -3’ | 145 | 60 ºC |
| TLR4 | F:5’ – AGTTGATCTACCAAGCCTTGAGT -3’  R:5’- GCTGGTTGTCCCAAAATCACTTT -3’ | 94 | 60 ºC |
| TLR7 | F:5’ – CACATACCAGACATCTCCCCA -3’  R:5’- CCCAGTGGAATAGGTACACAGTT -3’ | 92 | 60 ºC |
| TLR8 | F:5’ – GACTACAGGAAGTTCCCCAAAC -3’  R:5’- ATACCGGGATTTCCGTTCTGG -3’ | 169 | 60 ºC |
| TLR9 | F:5’- AATCCCTCATATCCCTGTCCC -3’  R:5’- GTTGCCGTCCATGAATAGGAAG -3’ | 104 | 60 ºC |

Supplementary table 3: mRNA TLR3 expression in the peripheral blood (polymorphonuclear and mononuclear cells) of patients with MILD COVID-19 and SEVERE COVID-19

|  | Polymorphonuclear Cells |  | Mononuclear Cells |  |  |
| --- | --- | --- | --- | --- | --- |
|  | Mean | StDev | Mean | StDev | p*  (PMN vs MONO) |
| MILD | 2.06 | 2.14 | 1.94 | 2.37 | 0.945 |
| SEVERE | 1.82 | 0.84 | 1.77 | 1.68 | 0.931 |
| p*  (MILD vs SEVERE) | 0.821 |  | 0.866 |  |  |

Variables are expressed as mean (standard deviation). * P values were calculated using the unpaired two-samples t-test in R software (version 4.0.3 for macOS)
